# Computer Modeling for Single-Screw Extrusion of Wood–Plastic Composites

**DOI:** 10.3390/polym10030295

**Published:** 2018-03-09

**Authors:** Krzysztof Wilczyński, Kamila Buziak, Krzysztof J. Wilczyński, Adrian Lewandowski, Andrzej Nastaj

**Affiliations:** Polymer Processing Department, Faculty of Production Engineering, Warsaw University of Technology, 02-524 Warsaw, Poland; kami.buziak@gmail.com (K.B.); wilczynski_k@wp.pl (K.J.W.); a.lewandowski@wip.pw.edu.pl (A.L.); a.nastaj@wip.pw.edu.pl (A.N.)

**Keywords:** modeling, extrusion, wood–plastic composites

## Abstract

Experimental and theoretical studies have been performed on the single-screw extrusion of wood–plastics composites. Experimental research has been carried out on the flow and melting of polypropylene (PP)-based composites with different wood flour (WF) content in the single-screw extruder. Based on these experimental observations, elementary models of the process phenomena have been proposed and a global model of the process has been developed. This global computer model includes the models of solid conveying, melting dependent on the wood flour content, melt flow in the screw, and melt flow in the die. 3-D non-Newtonian finite element method (FEM) screw pumping characteristics have been applied to model the melt flow in the metering section of the screw. The model predicts the extrusion output, pressure and temperature profiles, melting profile, and power consumption. The model has been validated experimentally. An effect of material slip on the extruder operation has been discussed including both slipping in the screw/barrel surfaces and in the extruding die.

## 1. Introduction

Wood–plastic composites (WPC) are made up of thermoplastics and wood flour or fiber. Both are polymer-based materials, but synthetic and natural, respectively. Thermoplastics are high-molecular-weight materials forming a continuous matrix that surrounds the wood component. These matrix materials are usually low-cost common thermoplastics that flow easily when heated, which translates to processing flexibility when wood flour or fiber is composed with them. Thermoplastics shrink and swell, but absorb only a small amount of moisture, and can be an efficient barrier to moisture penetrating into the composite. Wood flour or fiber is composed of natural polymers such as lignin, cellulose, etc., and has very different properties from the synthetic polymers. Wood is less expensive and stronger than synthetic polymers, and it may be a useful filler or reinforcement. The relatively low density of these natural fillers is also an important advantage. Wood does not shrink and swell substantially with temperature, but easily absorbs moisture which leads to biodegradation when not protected.

WPC exhibit high durability, relatively high stiffness and strength, and low price compared with other competitive materials. They are resistant to water and weather; and can be used for various outdoor applications. The WPC market has increased considerably in the last few years which is mainly driven by the growing building and construction market and by the automative industry [1]. The most commonly used thermoplastics for WPC are high-density polyethylene (HDPE), polypropylene (PP), and polyvinyl chloride (PVC) [1,2,3,4,5,6].

Worldwide WPC production has increased from 2.43 million tonnes in 2012 to 3.83 million tonnes in 2015, and it keeps growing. Although North America was the world’s production leader in 2012 (1.1 million t), ahead of China (900,000 t) and Europe (260,000 t), it was expected that China (with 1.8 million t) would have overtaken North America (1.4 million t) in 2015. European production was to increase by around 10% per year and reach 350,000 tonnes in 2015 [1].

Currently, due to extensive use of WPC in the form of profiles, the basic technology for processing these materials is extrusion. Extrusion is the most important technology in the polymer processing industry. It is broadly used for profile production (pipes, films, sheets, etc.), as well as for specialty polymer processing operations, such as compounding, pelletizing, mixing, devolatilization, chemical reactions, etc.

Extrusion of WPC differs considerably from plastics extrusion; this results from the different thermal and rheological properties of these highly filled materials, different structure, etc. Very limited research has been carried out on the rheology and extrusion of wood–plastic composites. Fundamental research has been performed by Xiao and Tzoganakis [7,8,9,10] as well as by Li and Wolcott [11,12,13]. Important contributions to this subject have been also delivered by Oksman Niska and Sain [5], Vlachopoulos et al. [14,15,16], and Zolfaghari et al. [17].

Now, it is known that WPC are pseudoplastic and viscoelastic materials. The viscosity of these materials decreases with an increase of shear rate and temperature; and increases with an increase of filler content. A decrease of elasticity with an increase of filler content has been also observed (Li and Wolcott [11,12,13], Mohanty et al. [3], Klyosov [4], Oksman Niska and Sain [5]). WPC exhibit yield stress and wall slip during extrusion, as reported by Xiao and Tzoganakis [7], Li and Wolcott [11], and Hristov et al. [14]. The wall slip velocity is dependent on the wood filler content and shear rate. With an increase of shear rate the slip velocity increases, which leads to the plug flow (Vlachopoulos and Hristov [15]). Also, an increase in wood content promotes the plug flow (Zolfaghari et al. [17]).

Recently, an interesting study has been performed by Laufer et al. [18] for WPC with different matrices and different types and contents of wood fillers. It has been concluded that shear thinning behavior of the WPC melts can be well described by the power law, and the consistency index of the power law well correlates with the wood volume fraction. The concept of equivalent inner shear rate has been developed, and on this basis the shear thinning behavior of WPC can be estimated with good accuracy, taking into account the wood volume fraction.

Designing the extrusion process of wood–plastic composites requires some knowledge about the flow mechanism of these highly filled materials in the extruder and in the die. Unfortunately, current theories do not predict the extrusion process course of these composites, e.g., material melting, pressure, and temperature distribution [8,10,16].

A few melting studies have been performed by Xiao and Tzoganakis [7,8,10] for extrusion of high-density polyethylene (HDPE) composite, and experimental data has been compared to simulations using commercial software. However, the predictions of pressure were not consistent with those measured.

Recently, Wilczyński et al. [19] have carried out extensive experimental studies on extrusion of a polypropylene (PP) composite. It was observed that material transport and melting were strongly dependent on the material composition and process parameters. The contiguous solids melting mechanism (CSM), known as a Tadmor mechanism [20], was not seen for composites of higher wood flour (WF) content. However, it was seen for composites of lower WF content of up to 50%. Several steps of the process were distinguished: first, compacting of the material in the solid conveying section close to the active flight and spreading to the rest of material; then, melting strongly dependent on the material composition; and finally shear or plug flow with possible slipping at the walls in the melt conveying section.

Up to the present, there is not any global model of WPC extrusion that would include solid conveying, melting, and melt flow. The state of the art of the extrusion of filled polymers has been recently discussed by Ariffin and Ahmad [21], and that of modeling of polymer extrusion by Wilczyński et al. [22]. In this paper, modeling of single-screw extrusion of wood–plastic composites is discussed, and an original computer model of the process is proposed and validated experimentally.

The basis for modeling the extrusion process is a knowledge of a material flow mechanism in the machine, i.e., solid transport, melting, and melt flow. The melt flow is quite well understood, but only for viscous flows. Viscoelastic flows are much less well understood. The flow of filled plastics that usually exhibit yield stress and wall slip is also poorly understood. Thus, global extrusion models are generally limited to viscous flows. To produce global models for wall-slipping materials it is necessary to develop models both for a plasticating unit and for an extrusion die [23,24,25,26].

Solids conveying and melting are much less understood and investigated, especially for advanced polymeric materials such as polyblends and composites. It is important to note that a melting model is always fundamental for the development of the global model of a process.

**Figure 1:**
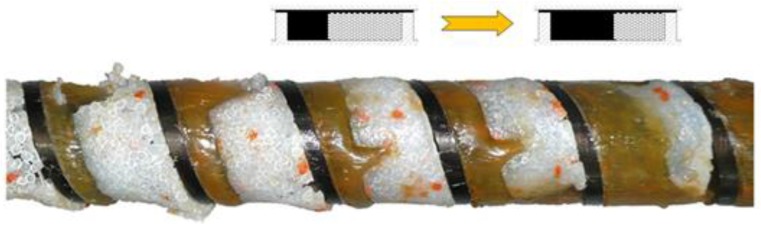
Classical melting mechanism of polymers in the single-screw extruder on an example of extrusion of polypropylene (PP). Reprinted with permission from [29]. Copyright Wiley 2012.

Melting in single-screw extruders has been widely studied and modeled. The first observations were reported by Maddock [27] and Street [28]. Their studies were based on the rapid cooling of an extruder, removing the screw and the polymer strip that was cross sectioned. This technique is called the “screw pulling-out technique”. In the melting section, a melt layer was seen along the barrel, and a melt pool close to the active flight. These observations were confirmed by many other researchers, e.g., by Wilczyński et al. [29] (Figure 1).

Tadmor [30,31] and Tadmor and Klein [20] first developed a model for the melting of polymers in single-screw extruders, and finally the first global model of the single-screw extrusion process. According to this model, a melt layer is formed between the barrel and solid, and molten polymer is scraped off by the transverse flow and accumulates at the active flight of the screw. The solid bed is gradually decreased by the combined effect of heating from the barrel and viscous dissipation in the melt. Several melting models were later developed and have been recently discussed by Altinkaynak et al. [32], and global extrusion models have been built up, e.g., by Agur and Vlachopoulos [33], Vincelette et al. [34], Potente et al. [35], and Wilczyński [36].

Contrary to the melting in single-screw extruders, the studies on melting in twin-screw extruders, both co-rotating and counter-rotating, appeared much later in the literature. Several melting models have been built up for co-rotating extruders, e.g., by Bawiskar and White [37,38], Sakai [39], Potente and Melish [40], Gogos et al. [41], and Vergnes et al. [42,43], and a few global models have been developed, e.g., by Bawiskar and White [44], Vergnes et al. [45] and Potente et al. [46].

Limited studies have been performed on melting in counter-rotating extruders, e.g., by Wilczyński and White [47], and Wilczyński et al. [48]. A melting model and the first global models have been developed by Wilczyński and White [49], Wilczyński et al. [50,51], and Jiang et al. [52].

The literature on starve-fed single-screw extrusion is extremely limited. Recent experimental studies performed by Wilczyński et al. [29] revealed the mechanism of melting in starve-fed single-screw extruders, and then the authors developed a mathematical model of melting of polymers in these machines [53], and finally a global model of the process [54].

Summarizing, reviews on the modeling of polymer extrusion have been recently presented by Wilczyński et al. [22], Malik et al. [24], Ilinca and Hetu [55], and Teixeira et al. [56]. A few basic monographs have also taken up this problem, e.g., by White and Potente [57], Tadmor and Gogos [58], and Rauwendaal [59].

## 2. Experimental

An instrumented single-screw extruder was used in the study, equipped with a barrel of diameter D = 45 mm and a length/diameter ratio (L/D) = 27. The all-purpose three-sectional screw was applied. It has feeding, compression, and metering sections with length/diameter ratios equal to (L/D)_F_ = 10.78, (L/D)_C_ = 7.11, and (L/D)_M_ = 9.11. The compression ratio, which is defined as a ratio of the channel depth (H_F_) in the feeding section to the channel depth in the metering section (H_M_), i.e., CR = H_F_/H_M_, was equal to CR = 2.66 (H_F_ = 8 mm, H_M_ = 3 mm). The die for extruding rods of diameter D = 5 mm was applied. Pressure, temperature, and power consumption were measured in the study. A “screw pulling-out technique” was applied to investigate the material flow and melting mechanism. This technique is described elsewhere [29]. The screw speed was set at N = 20 rpm, N = 50 rpm, N = 80 rpm, and the temperature at T_I_ = 160 °C, T_II_ = 180 °C, T_III_ = 190 °C, T_IV_ = 190 °C in the four barrel sections (of equal length) and T_DIE_ = 180 °C in the die. All these parameters have been collected in Table 1 and Table 2.

Various polypropylene (PP)-based composites were used in the study (manufactured by Beologic). These were WPC of WF/PP compositions equal to 60/40, 50/50, 40/60, and 30/70. We used (i) WPC 60/40 PP ext 044/0-400, (ii) WPC 50/50 PP copo inj 4, (iii) WPC 70/30 PPC ext 101/0-400, and PP copo inj 2. The composites with WF/PP content equal to 40/60 and 30/70 were obtained by blending WPC 70/30 with PP in a single-screw extruder using a granulating die. A small portion of the material (3%) was colored with 2% Ramafin-Orange CEK071 dye-stuff to distinguish between solid and melt. The material was dried at 80 °C for 4–6 h.

Polypropylene has density 0.9 g/cm^3^, melt flow index MFI = 25 g/10 min (230 °C, 2.16 kg), and a melting point of about 160 °C. The WPC composites have density 1.1–1.3 g/cm^3^, bulk density 0.4–0.6 g/cm^3^, and mass flow rate index MFR = 3 g/10 min (200 °C, 5 kg).

**Figure 2:**
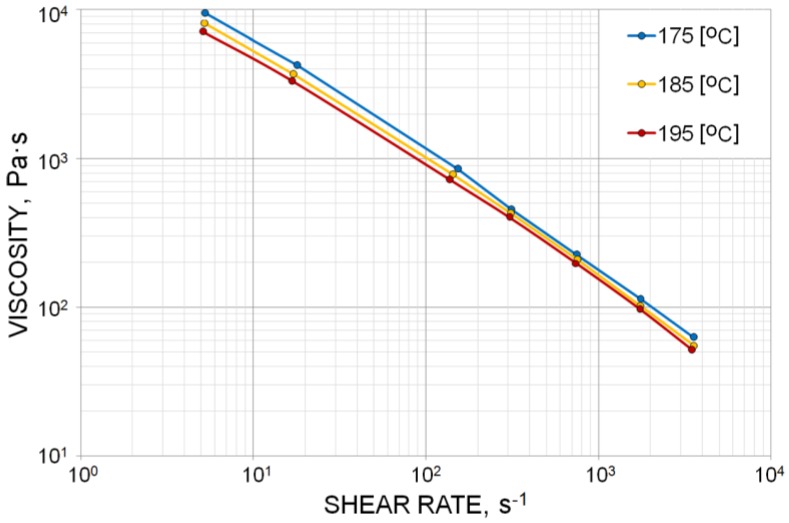
Viscosity curves of wood–plastic composite (WPC) 50/50 polypropylene (PP) copo inj 4.

Viscosity curves were determined for WPC 50/50 PP copo inj 4. Rheometric tests were carried out using a high-pressure capillary rheometer Rheograph 6000 (from Goettfert, Buchen, Germany) operating on the principle of constant shear rate. The measurements were performed in the temperature range 175–195 °C and the shear rate range 5–3500 s^−1^. Rabinowitsch and Bagley corrections were applied in rheological calculations. The viscosity characteristics are depicted in Figure 2. The viscosity curves present a typical non-Newtonian pseudoplastic behavior with decreasing viscosity with an increase of shear rate.

The mass flow rate index (MFR) was additionally determined for this composite using a Melt Indexer MI-2 (from Goettfert). The tests were carried out at 190 °C, and two loads were applied—High Load (HL = 21.6 kg) and Low Load (LL = 10 kg). We obtained MFR_HL_= 38 g/10 min, and MFR_LL_ = 4 g/10 min.

## 3. Results

**Figure 3:**
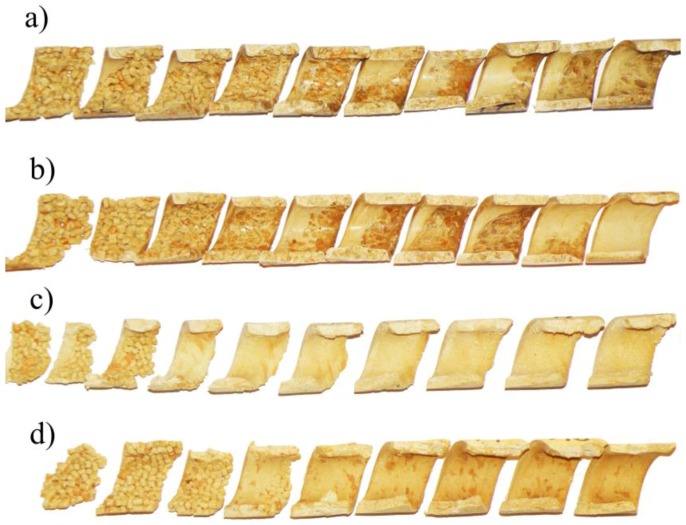
The effect of wood flour (WF) content on the material flow in the screw channel (samples of the material of different wood content removed from the screw, obtained by extrusion at the screw speed N = 50 rpm): (**a**) WF = 30%, (**b**) WF = 40%, (**c**) WF = 50%, (**d**) WF = 60%.

**Figure 4:**
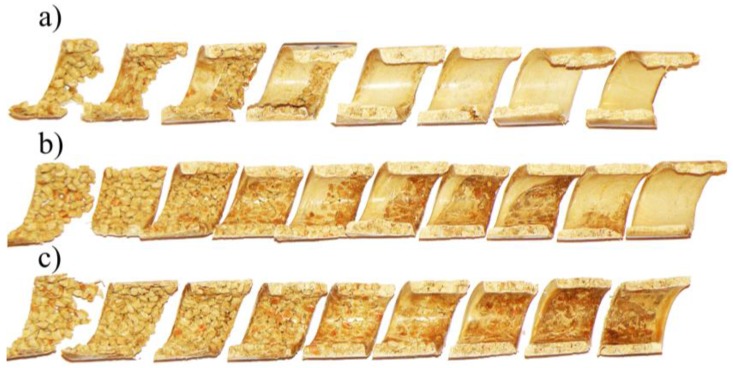
The effect of screw speed on the material flow in the screw channel (samples of the material of 40% WF content removed from the screw): (**a**) N = 20 rpm, (**b**) N = 50 rpm, (**c**) N = 80 rpm.

**Figure 5:**
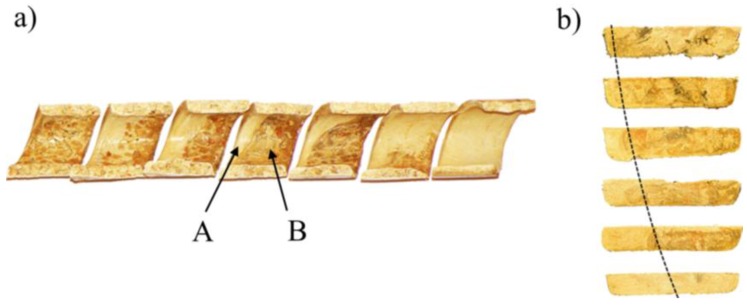
Mechanism of melting of wood–plastic composite (WPC) in the single-screw extruder: (**a**) a view from the screw side (WF = 40%), (**b**) cross sections (WF = 30%); A—molten material, B—solid material.

**Figure 6:**
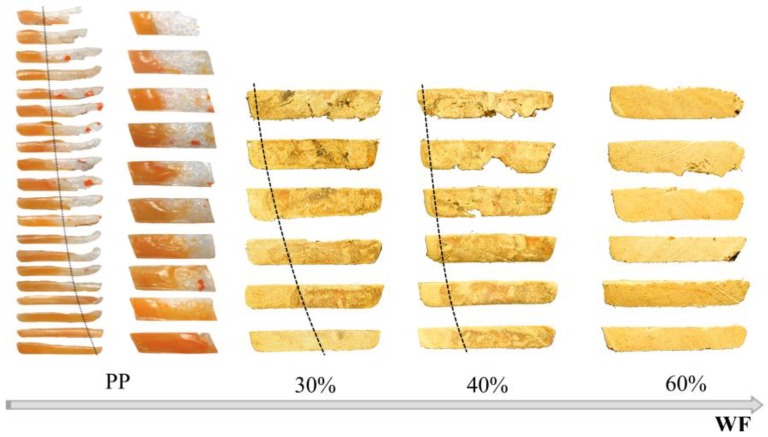
The effect of wood flour (WF) content (30–60%) on the melting mechanism (presented as cross sections) of wood–plastic composites (WPC) in the single-screw extruder compared with [29] melting of neat polypropylene (PP). Reprinted with permission from [29]. Copyright Wiley 2012.

The main goal of experimental investigations was to learn the mechanism of flow and melting of the studied composites as the physical basis for modeling. Sample results of the tests are presented in Figure 3, Figure 4, Figure 5 and Figure 6.

It was observed that in the vicinity of the hopper the material forms a loose structure which gradually thickens starting from the active flight of the screw, and the material starts to melt after being compacted. The effect of wood flour (WF) content on the material structure and flow in the screw channel is shown in Figure 3.

It is clearly visible that with a lower WF content there is a classical melting mechanism with accumulation of the molten material (melt pool) at the active flight of the screw. With higher WF content this mechanism does not occur. This can be explained by the small amount of molten material being not enough to form the melt pool, and also by insufficient pressure in this area which is necessary to initiate forming the melt pool. It can be predicted that, in this case, the molten material penetrates into the unmolten layer and melting has a one-dimensional character, proceeding from the barrel to the core of the screw. A similar mechanism was observed when extruding plastics in powdered form [60], as well as in the case of our previous tests [19]. After melting was completed, shear melt flow or plug flow with slipping at the walls occurred. It was observed that an increase in wood content promoted existence of the plug flow.

The influence of the screw rotational speed on the material flow is shown in Figure 4. It can be seen here that with an increase of the rotational speed of the screw, melting begins later, i.e., further away from the hopper, and closer to the die. Melting also seems to be slower since the residence time of the material in the extruder is shorter.

The classical mechanism of melting of wood–plastic composites (WPC) in the single-screw extruder is shown in Figure 5, in the side view of the screw and in the cross section.

Figure 6 presents cross-sectional views of composites with different wood flour (WF) content (in the melting section) compared with cross-sectional views of neat polypropylene (PP). The similarity is obvious, although with an increase in the WF content the melt pool decreases.

During the tests, the pressure and flow rate were measured; these were compared with the simulation data and are presented in Table 3. The influence of the rotational screw speed on the material flow rate (i.e., the extrusion throughput) and extrusion pressure (i.e., the die pressure) was quite obvious. As the screw speed increased, the flow rate and the die pressure increased. However, this dependence was not proportional but rather weaker.

## 4. Modeling

**Figure 7:**
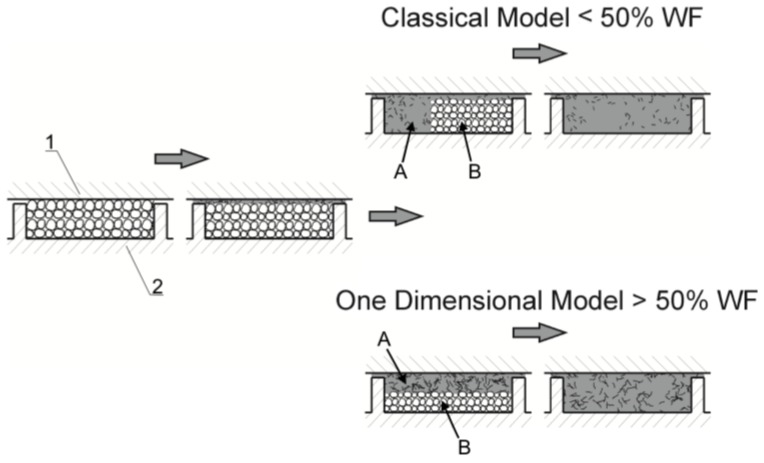
Geometrical model of melting mechanism of wood–plastic composites (WPC) in the single-screw extruder with dependence on wood flour (WF) content: 1—barrel, 2—screw, A—molten material, B—solid material.

On the basis of the performed research, a mechanism for flow and melting of wood composites in a single-screw extruder was proposed; this mechanism is depicted in Figure 7.

An extrusion process is the result of interaction between the extruder and the die. Extrusion modeling must take this interaction into account. It is not possible to consider the phenomena occurring in the extruder in isolation from the flow of material in the die. The flow of material in the die is relatively simple and well described (for viscous flows), although not in the case of the wood composites tested in this work. The flow of material in the extruder is much more complex, since it involves transporting the material in the solid state, melting, and flow of the molten material. The description of this flow is difficult even in the case of neat polymers, and especially difficult and very poorly understood in the case of composite materials.

In the conventional extrusion process (without dosing), the flow rate of the material is not determined by the extruder operator, but it results from the cooperation of the extruder–die system. Conditions of this cooperation are determined by the operating point of the extruder, which determines the extrusion throughput (polymer flow rate), and the extrusion pressure (die pressure). The basis of the calculation algorithm is to solve the problem of determining the flow rate and pressure distribution in the plasticating unit and the die. This problem can only be solved using the iterative calculation procedure in which the compliance of the pressure increase in the plasticating unit is tested with the pressure drop in the die. This is classical approach presented, e.g., in [36].

The global extrusion model is a series connection of elementary models describing the material flow in the plasticating unit of the extruder; that is, models describing transport of solid material, melting, and flow of molten material, and then the melt flow in the die.

Since the flow rate during extrusion is constant, the process can be considered as a series of separate elementary segments having the same mass flow rate (G), and locally constant material and process parameters. This is the lumped parameter approach which is sufficient for engineering computations. The process parameters, e.g., pressure, temperaturę, or solid content, at the beginning of each segment are equal to the process parameters at the end of the previous segment, i.e.,

p_i_in_ (z) = p_i-1_out_ (z) (1)

where p_i_in_ (z) is the process parameter (e.g., pressure or temperature) at the beginning of the *i*th segment, p_i-1_out_ (z) is the process parameter at the end of the (*i* − 1)th segment, and z is the location of the segment Δz along the screw channel length.

The global extrusion model presented here contains some elements of our previous developments in the range of solid conveying [36], and introduces new solutions for melting and melt flow (applying 3-D screw pumping characteristics).

In summary, the model consists of the following elementary models:

– Solid conveying, where delay in melting is allowed;
– Melting 1-D or 2-D, depending on the wood flour content;
– Melt conveying in the plasticating unit using 3-D finite element method (FEM)-based screw pumping characteristics;
– Melt flow in the die 1-D or 3-D FEM.

**Figure 8:**
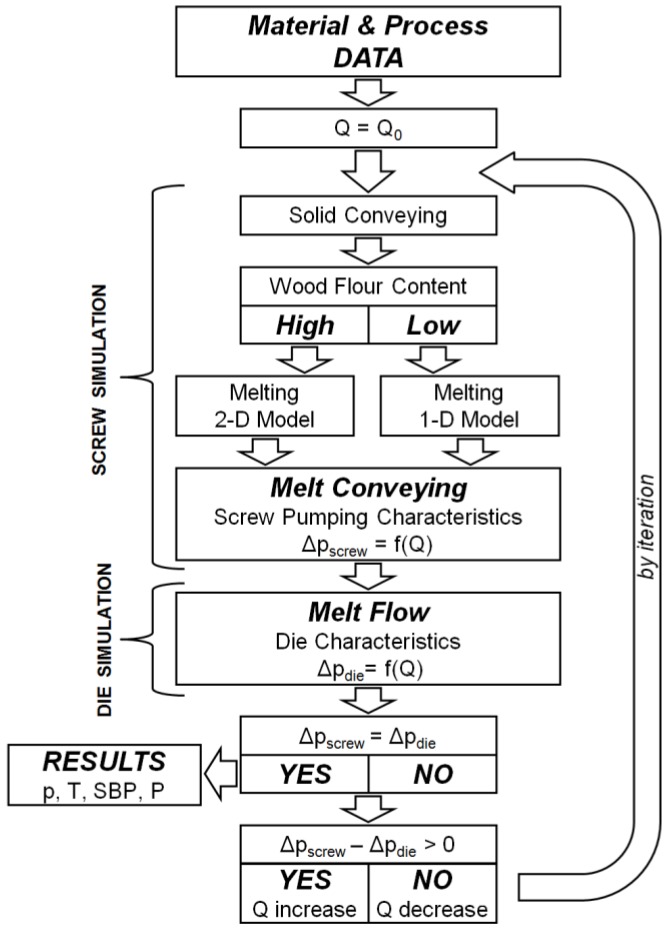
Scheme of computations: Q—flow rate, Q_0_—initial flow rate, p—pressure, Δp_screw_—total pressure increase in the screw, Δp_die_—total pressure drop in the die, T—temperaturę, SBP—melting profile (Solid Bed Profile), P—power consumption.

The scheme of computations is depicted in Figure 8. The computations start assuming some initial value of the flow rate, e.g., equal to the drag flow rate. Then the process is simulated with dependence on the wood flour content (WF) according to 1-D or 2-D melting model. Next, 3-D screw pumping characteristics are applied for modeling of the melt conveyance, and the pressure at the screw exit is finally computed. This pressure is compared with the pressure drop in the die (in general computed as 1-D flow), and convergence is checked. Depending on the result (negative or positive) the flow rate is changed (increased or decreased) and computations are repeated until convergence is reached.

A one-dimensional melting process (1-D model) can be described on the basis of the heat balance on the interface of the solid and melt, and of the mass balance in the melt and solid. In order to make such a balance, one should determine the velocity and temperature profile in the melt film and the temperature profile in the solid.

According to the heat balance, the heat flux used to melt the material is equal to the difference between the heat flux from the film into the melt/solid interface and the heat flux from the interface into the solid bed. This balance does not differ from the heat balance of the two-dimensional classic approach (2-D model) [36,58].

However, the mass balance in the melt and solid differs since the solid bed width is constant in this case and equal to the screw channel width. The material is melted and the volume of melt increases; however, the melt pool at the active flight of the screw is not formed. An increase in melt volume, i.e., in film thickness, decreases the dissipation since the shear rate decreases when the film thickness increases. This causes slower melting.

Global modeling of the extrusion process requires iterative, multiple calculations (sometimes hundreds of iterations). Therefore, the use of time-consuming calculations using the finite element method (FEM) is not used here. However, you can develop the so-called flow characteristics based on FEM calculations, and then implement them in the form of regression models to the global process model. A good accuracy of the calculation is then obtained with reasonable calculation time.

These flow characteristics, i.e., screw pumping characteristics, are defined as functions of dimensionless flow rate and dimensionless pressure gradient:

Q* = f (Δp*)(2)

where Q* is the dimensionless flow rate, and Δp* is the dimensionless pressure gradient.

This concept has been recently applied by the authors for starve-fed and flood-fed single-screw extrusion with conventional screws [54] and with mixing screws [61,62], as well as for counter-rotating twin-screw extrusion [63,64].

In this study, we model single-screw extrusion with conventional screws, and in this case the screw pumping characteristics are defined as

Q* = 2Q/WHV_bz_, (3)

Δp* = H^n+1^sin φ/6mV_bz_^n^·(Δp_c_/L_f_), (4)

where Q is the flow rate, W is the screw channel width, H is the screw channel depth, V_bz_ = πD_b_Ncos φ is the *z*-component of the circumferential velocity at the barrel, D_b_ is the barrel diameter, N is the screw speed, φ is the helix angle, Δp_c_ is the pressure change, L_f_ is the screw length of the pressure change, m is the consistency coefficient, and n is the power-law exponent.

**Figure 9:**
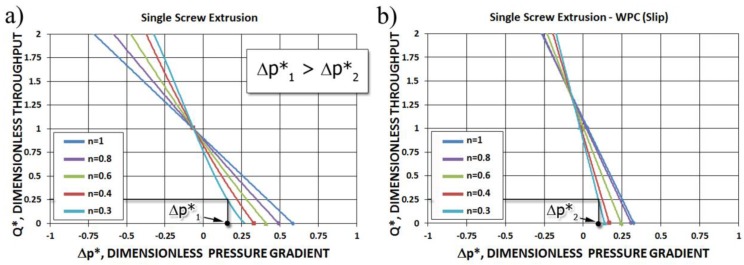
Screw pumping characteristics: (**a**) without slip, (**b**) with slip (see Section 6).

These characteristics are depicted in Figure 9a for different values of power-law exponent.

## 5. Simulations

Simulations were performed for the range of experimental material and processing data. These parameters are collected in Table 1 and Table 2.

The viscous flow properties were modeled using the Klein rheological equation [65]:

(5) $$\ln\eta =A_{0}+A_{1}\ln\dot{\gamma }+A_{11}\ln^{2}\dot{\gamma }+A_{12}\ln\dot{\gamma }T+A_{2}T+A_{22}T^{2}$$

where η is the viscosity, Pa·s; $\dot{\gamma }$ is the shear rate, s^−1^; T is the temperaturę, °C; and A_0_, A_1_, A_11_, A_12_, A_2_, A_22_ are the model parameters (A_0_ = 12.469780638, A_1_ = −0.8345507, A_11_ = −0.017832191, A_12_ = 0.001331159, A_2_ = −0.008413991, A_22_ = −0.000025745).

**Figure 10:**
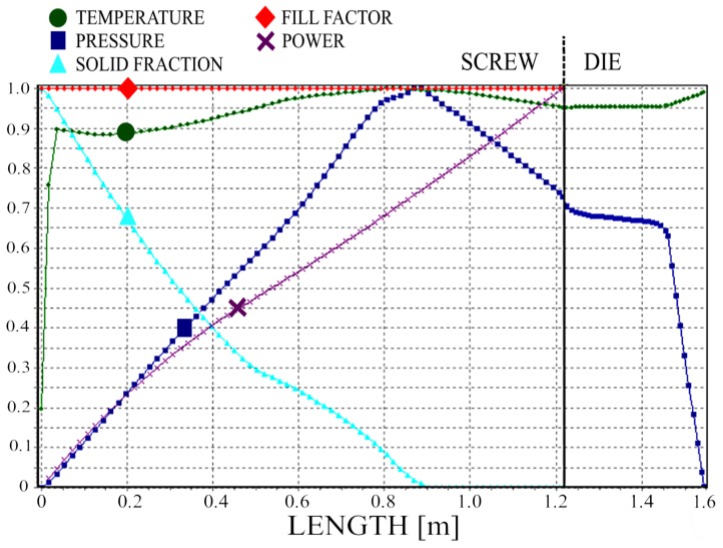
Dimensionless overall extrusion process characteristics, N = 80 rpm.

**Figure 11:**
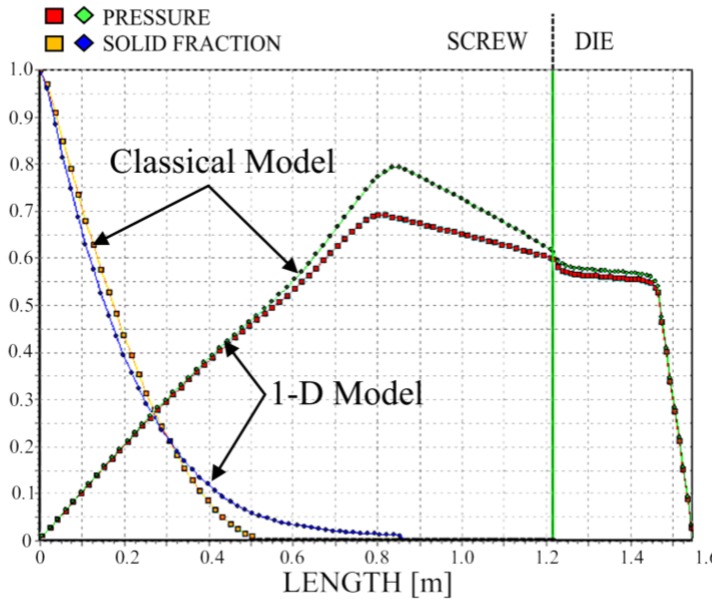
Solid bed profile and pressure profile for 1-D and 2-D simulations, N = 20 rpm.

**Figure 12:**
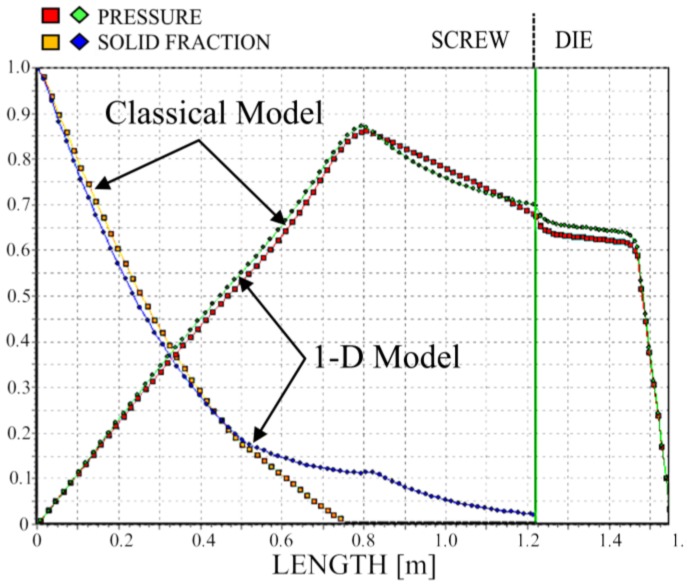
Solid bed profile and pressure profile for 1-D and 2-D simulations, N = 50 rpm.

**Figure 13:**
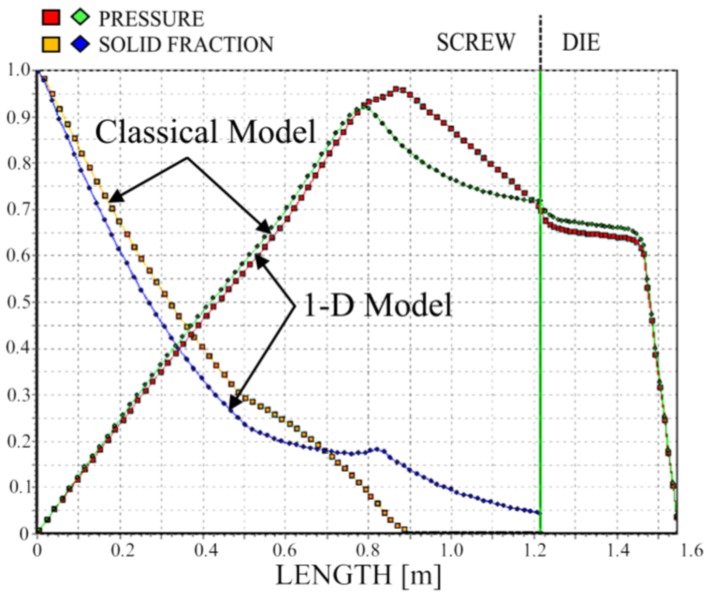
Solid bed profile and pressure profile for 1-D and 2-D simulations, N = 80 rpm.

Simulation results (dimensionless) are depicted in Figure 10, Figure 11, Figure 12 and Figure 13. Overall extrusion characteristics are depicted in Figure 10. These include pressure and temperature profiles, melting profile, i.e., solid bed profile (SBP), and power consumption profile, as well as the fill factor profile for starve-fed extrusion which determines the extent of filling of the screw.

We simulated the process using two different models of melting—the classical two-dimensional model and a one-dimensional model. The results are depicted in Figure 11, Figure 12 and Figure 13. It is clearly seen that melting according to the 1-D model is substantially slower; however, the impact on the pressure profile is not significant.

**Figure 14:**
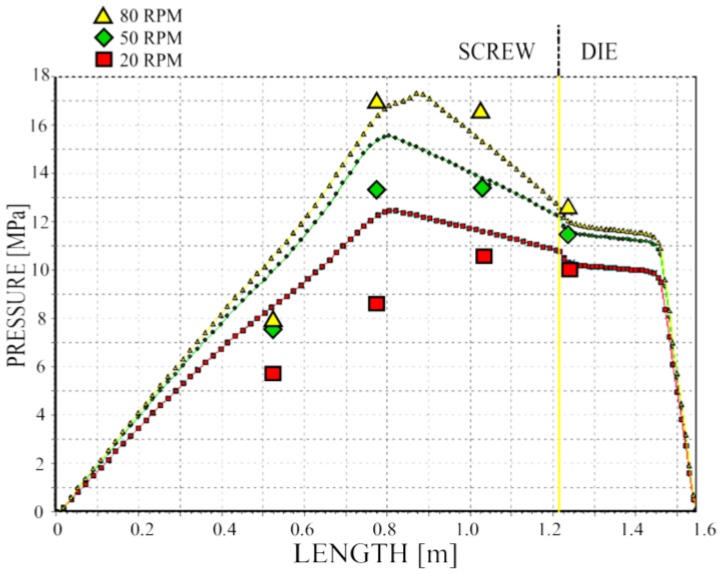
Validation of pressure computation.

We validated pressure computations, and the results of validation are depicted in Figure 14. The pressure profile is well predicted; however, in general, it is overestimated.

## 6. Modeling of Slip Effects

Wood–plastic composites are wall-slipping materials and this fact has not been considered in modeling in the previous sections. This may be one of the causes of discrepancies between simulations and experimentations. Detailed study on the problem of global modeling of the extrusion process with slip effects will be presented elsewhere [66]. In this paper, some crucial aspects of this issue are presented.

ANSYS Polyflow CFD software [67] has been used for simulations of the extrusion process with slip effects, and the generalized Navier’s law has been applied for slip modeling. This law can be expressed in the following way:

(6) $$f_{s}=F_{\mathrm{slip}}\left(V_{\mathrm{wall}}-V_{s}\right){\left|V_{s}-V_{\mathrm{wall}}\right|}^{e_{\mathrm{slip}}-1}$$

where f_s_ is the shear stress, V_s_ is the tangential velocity of the fluid, V_wall_ is the tangential velocity of the wall, and F_slip_ and e_slip_ are the material parameters (F_slip_ = 0 denotes full slip conditions, i.e., f_s_ = 0, and e_slip_ = 1 denotes linearity of the law).

The constitutive equation of power-law fluid has been used in this study for flow modeling, and is expressed as

(7) $$\tau \;=m{\dot{\gamma }}^{n}$$

where τ is the shear stress, $\dot{\gamma }$ is the shear rate, m is the consistency coefficient and n is the power-law exponent.

The parameters of the model were m = 30,111.9 Pa·s^n^ and flow index n = 0.249.

**Figure 15:**
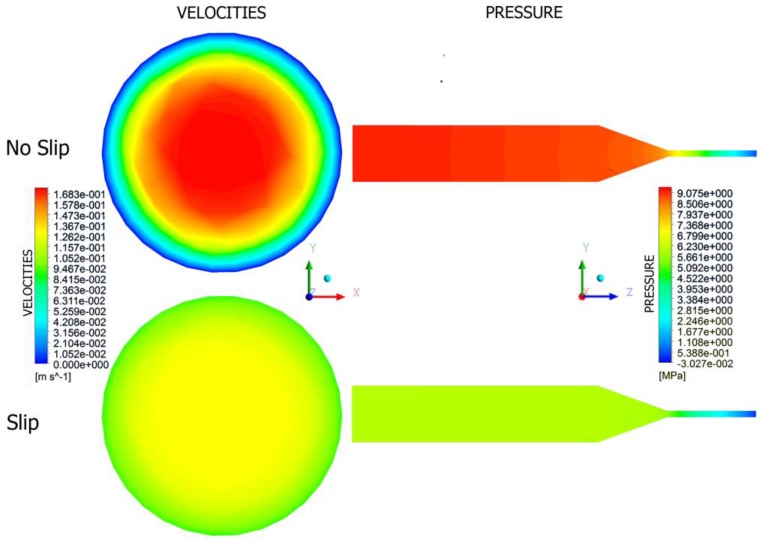
Die flow simulations.

**Figure 16:**
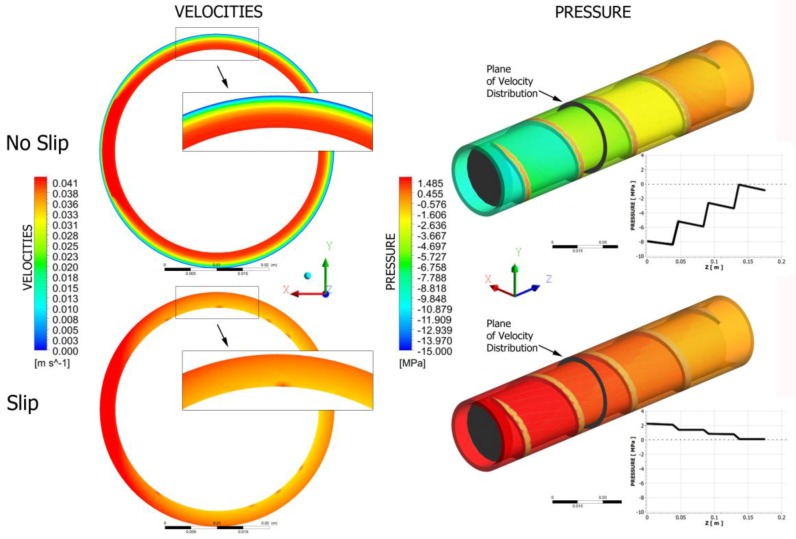
Screw flow simulations.

Fully three-dimensional non-Newtonian simulations have been performed of the flow with slip effects both in the extruder (on the screw and barrel surfaces) and in the die. This allowed global study of operation of the extrusion process of wall-slipping polymers. Examples are depicted in Figure 15 and Figure 16. It is clearly seen that the velocity profile drastically changes and pressure substantially drops when slipping increases, both in the die and in the extruder.

Screw pumping characteristics at different slip conditions (with slip and without slip) are shown in Figure 9 for Newtonian and non-Newtonian cases. Decreasing the power-law exponent, which increases the non-Newtonian character of the flow, importantly reduces the screw pumping capacity (for positive pressure gradient). It is also clearly seen that slipping decreases the pressure gradient both when positive or negative.

**Figure 17:**
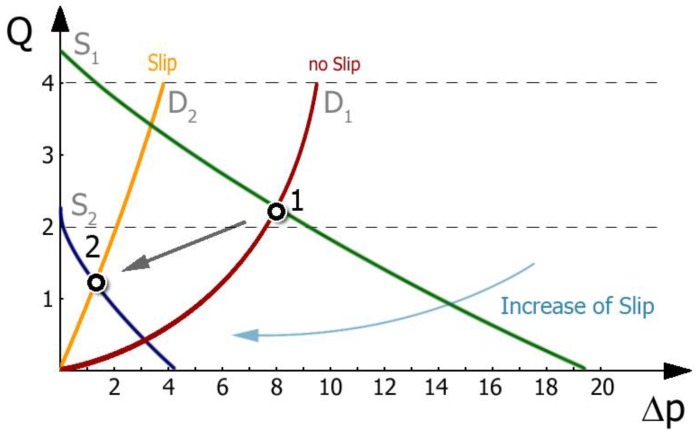
Extruder operating characteristics: Q—flow rate, Δp—pressure change, S_1_, S_2_—screw characteristics, D_1_, D_2_—die characteristics.

Extrusion is a process of pushing of the polymer through a forming die, and can be considered as a series connection of the extruder and the die. Since the die exerts a resistance to flow, pressure is required to push the polymer through the die. One should know that the die pressure is caused by the die, and not by the extruder. The extruder simply produces pressure to push the polymer through the die. An extrusion process can be described by the extruder operating characteristics, which are determined by the screw characteristics and the die characteristics (Figure 17). Actual extrusion operating conditions are defined by the extruder operating point which results from an intersection of the screw characteristics and the die characteristics. This point determines the flow rate (throughput) and the extrusion pressure (die pressure).

Figure 17 shows the effect of slip conditions determined by the F_slip_ parameter of Navier’s law (6) on the extruder operating point. The starting point (1) has been obtained for low slip at the screw and no slip at the die. When slip at the die is allowed, and the slip at the screw increases, the operating point moves to lower flow rate and lower pressure, i.e., to the point (2). It can be concluded that slip at the screw and at the die have an important impact on the location of the extruder operating point—that is, on the flow rate and extrusion pressure. When slip at the screw increases, the flow rate and pressure decrease; when slip at the die increases, the flow rate increases and the pressure decreases.

## 7. Conclusions

Experimental research has shown that flow and melting of polypropylene (PP)-based wood composites are considerably different from those of neat polypropylene. In the initial part of the screw, the processed material forms a loose structure that gradually thickens and, after compaction, begins to melt. Melting depends on the content of wood flour (WF) in the material. In a simplification, it can be concluded that in the case of lower WF content classical 2-D melting takes place, while in the case of higher WF content 1-D melting is observed without a melt pool at the active flight of the screw. In this case, an increase in the melt volume, i.e., in the film thickness, decreases the dissipation since the shear rate decreases with an increase of the film thickness, and this causes slower melting.

A global computer model of single-screw extrusion of wood–plastic composites has been developed that describes solid conveying, melting dependent on the wood flour content, melt flow in the screw, and melt flow in the die. The model predicts the extrusion output, pressure and temperature profiles, melting profile, and power consumption. The model has been successfully validated experimentally; however, the pressure was overpredicted.

It has been concluded that slip at the screw and the die have an important impact on extruder operation. When slip at the screw/barrel increases, the extrusion output and pressure decrease, while when slip at the die increases, the extrusion output increases and the pressure decreases. We suggest an additional modeling study taking into account possible yield stress of the processed material.

## Figures and Tables

**Table 1:** Extruder screw geometry and dimensions.

Square-Pitch Screw	
Feed/compression/metering sections	10.78/7.11/9.11 turns
Barrel inside diameter, D_b_	45 mm
Screw pitch	45 mm
Channel depth in feed section, H_F_	8 mm
Channel depth in metering section, H_M_	3 mm
Flight width	5 mm

**Table 2:** Composite properties and processing variables.

**Extruder Operating Conditions**	
Barrel and die temperature profile	160/180/190/190/180 °C
Frequency of screw rotation	20, 50, and 80 rpm
**Material and rheological properties**	
Density	
- bulk, ρ_bulk_	500 kg/m^3^
- solid, ρ_s_	1080 kg/m^3^
- melt, ρ_m_	930 kg/m^3^
Polymer–barrel friction factor	0.25
Polymer–screw friction factor	0.15
Heat capacity	
- melt, Cp_m_	1850 J/(kgK)
- solid, Cp_s_	1160 J/(kgK)
Thermal conductivity—melt, k_m_	0.243 W/(mK)
Heat of fusion, λ	133,850 J/kg
Melting temperature, T_m_	125 °C
Viscosity, η (see Figure 2, Equation (5))	
- A_0_	12.469780638
- A_1_	−0.834550700
- A_11_	−0.017832191
- A_12_	0.001331159
- A_2_	−0.008413991
- A_22_	−0.000025745
where η [Pa∙s], γ̇ [s^−1^], T [°C]	

**Table 3:** Predicted versus measured process parameters.

	Experiment		Simulation (See Section 5)	
Screw Speed rpm	Throughput kg/h	Die Pressure MPa	Throughput kg/h	Die Pressure MPa
20	7.9	10	6.6	10.7
50	17.4	11	16.8	12.1
80	25.8	13	27.5	12.6
